# American Veterinary College

**Published:** 1895-09

**Authors:** 


### AMERICAN VETERINARY COLLEGE.

The session of this school commences October 1 and ends the latter part of the succeeding March, a period of twenty-two weeks, inclusive of holidays and examinations. We note no change in the matriculation examinations or requirements for graduation. The course of study has been more thoroughly graded than in previous years and the instruction imparted to the students in a more systematic manner. J. B. Stein, M.D., has been added to the teaching-staff as professor of physiology. Herbert Neher, D.V.S., has been added to the adjunct faculty as lecturer on external forms of the horse and zoötechny.

### SUGGESTIVE OF A SMILE.

THE desk of Post Veterinary Surgeon Hunter, of Fort Leavenworth, Kansas, bears two placards, giving the following terse information:

| THIS IS NOT THE POST HOSPITAL. | WE ARE OUT OF ALCOHOL, CAUSTIC, AND BLUE OINTMENT. |
|---|---|

Dr. Sesco Stewart asked a student to describe the "phenomena of an animal sweating blood." The student having missed the lecture on that subject, answered that he "believed it was caused by the animal being obliged to take an examination in helminthology."

### WORLD'S PROGRESS.

VETERINARIANS will be glad to learn of the early addition to veterinary literature of two excellent works, one on *General Pathology*, covering the various fields of causation of diseases, by that eminent student, careful investigator and writer, Professor H. J. Detmers, of Columbus, Ohio.

A second work on *Pathology* by Professor J. H. Huddleston, of the New York Veterinary College. Dr. Huddleston, while not so well known as yet to the veterinary profession, is one of the most earnest, interested instructors in veterinary science in the East. A patient, earnest, and thorough investigator, a fluent writer, and no doubt will make a very welcome contributor to our libraries.

The outbreak of rabies in Allegheny County has extended during the past few weeks, necessitating the destruction of a number of horses, mules, and cows, in addition to a large number of dogs. The owner of one dog, bitten by his rabid pet while endeavoring to chloroform it, has gone to the Pasteur Institute at New York City for treatment.

